# *Chlamydia psittaci* Meningitis, Chile, 2023

**DOI:** 10.3201/eid3210.251985

**Published:** 2026-10

**Authors:** Yazmin Ivana Pinos Garcia, Cristian Rosas, Josefa Estefania Elmohrez, Jose Vicente Gonzalez, Teresa Tapia, Juan Carlos Hormazabal

**Affiliations:** Universidad Austral de Chile, Facultad de Medicina, Osorno, Chile (Y.I. Pinos Garcia, J.E. Elmohrez); Hospital Base San Jose Osorno, Osorno (Y.I. Pinos Garcia, J.V. Gonzalez); SEREMI de Salud de Los Lagos, Osorno (C. Rosas); Institute of Public Health of Chile, Santiago, Chile (T. Tapia, J.C. Hormazabal)

**Keywords:** Chlamydia psittaci, psittacosis, meningitis/encephalitis, bacteria, zoonotic pathogens, zoonoses, Chile

## Abstract

We report a confirmed case of *Chlamydia psittaci* meningitis in Latin America, diagnosed in a 50-year-old farm worker from Chile. Diagnosis was established via cerebrospinal fluid quantitative real-time PCR and serology. The patient recovered after antimicrobial treatment. This case highlights the need to consider zoonotic pathogens in atypical meningitis.

*Chlamydia psittaci* is an obligate intracellular bacterium that causes psittacosis, a zoonotic infection typically transmitted through contact with infected birds, particularly Psittaciformes and poultry (*1*,*2*). In humans, psittacosis typically exhibits constitutional symptoms, including fever, chills, headache, and myalgia (*3*). Although widely associated with respiratory illness, the infection also can affect other organs, including the central nervous system (CNS), resulting in rare but serious manifestations, such as encephalitis and meningitis (*4*). We report a confirmed case of *C. psittaci*–associated meningitis in Latin America supported by molecular testing and serology.

## The Case

A 50-year-old male farm worker from Puerto Octay, southern Chile, sought treatment at a rural health center on August 14, 2023, with fever >38°C, myalgia, chills, headache, and painful swallowing. Health center clinicians established a diagnosis of tonsillitis and treated the patient with intramuscular benzathine penicillin. Ten days later, the patient returned with persistent fever and headache and was referred to San José Hospital in Osorno with suspected meningitis. The patient worked as a tractor operator on a dairy farm, where he had occasional direct contact with cattle through participation in calving assistance and milking activities. He reported an increased number of bovine abortions on the farm during the months preceding illness onset. Although he denied direct contact with birds, he reported the presence of wild parrots and domestic chickens on the farm.

At admission, we noted an erythematous pharynx but found the patient to be alert and oriented. Neurologic examination revealed normal cranial nerves, preserved motor function and tone, and no signs of meningeal irritation. Laboratory results revealed leukocytosis (11,400/mm^3^, 65% neutrophils), elevated C-reactive protein of 16.8 mg/L (reference value <5 mg/L), and mildly elevated liver enzymes. Cerebrospinal fluid (CSF) was clear and colorless, with 1,033 leukocytes/mm^3^ (56% mononuclear, 44% polymorphonuclear), protein 1.21 g/L, and glucose 50.1 mg/dL. We performed Gram stain and multiplex meningitis/encephalitis PCR panel of the CSF that included human parechovirus, adenovirus, cytomegalovirus, human enterovirus, parvovirus B19, Epstein-Barr virus, herpes simplex virus (HSV) 1 and 2, human herpesvirus 6 and 7, mumps virus, varicella-zoster virus, *Escherichia coli* K1, *Streptococcus pneumoniae*, *Hemophilus influenzae*, *Neisseria meningitidis*, *Streptococcus agalactiae*, and *Cryptococcus neoformans*; all tests were negative. HIV serology, cryptococcal antigen testing, and *Toxoplasma gondii* PCR also produced negative results. Cranial computed tomography revealed no abnormalities; magnetic resonance imaging detected white matter hyperintensities in both hemispheres at T2W, FLAIR, and DWI sequences (Figure), which have been described in meningitis caused by other pathogens, such as cytomegalovirus and *S. pneumoniae* (*5*).

**Figure:**
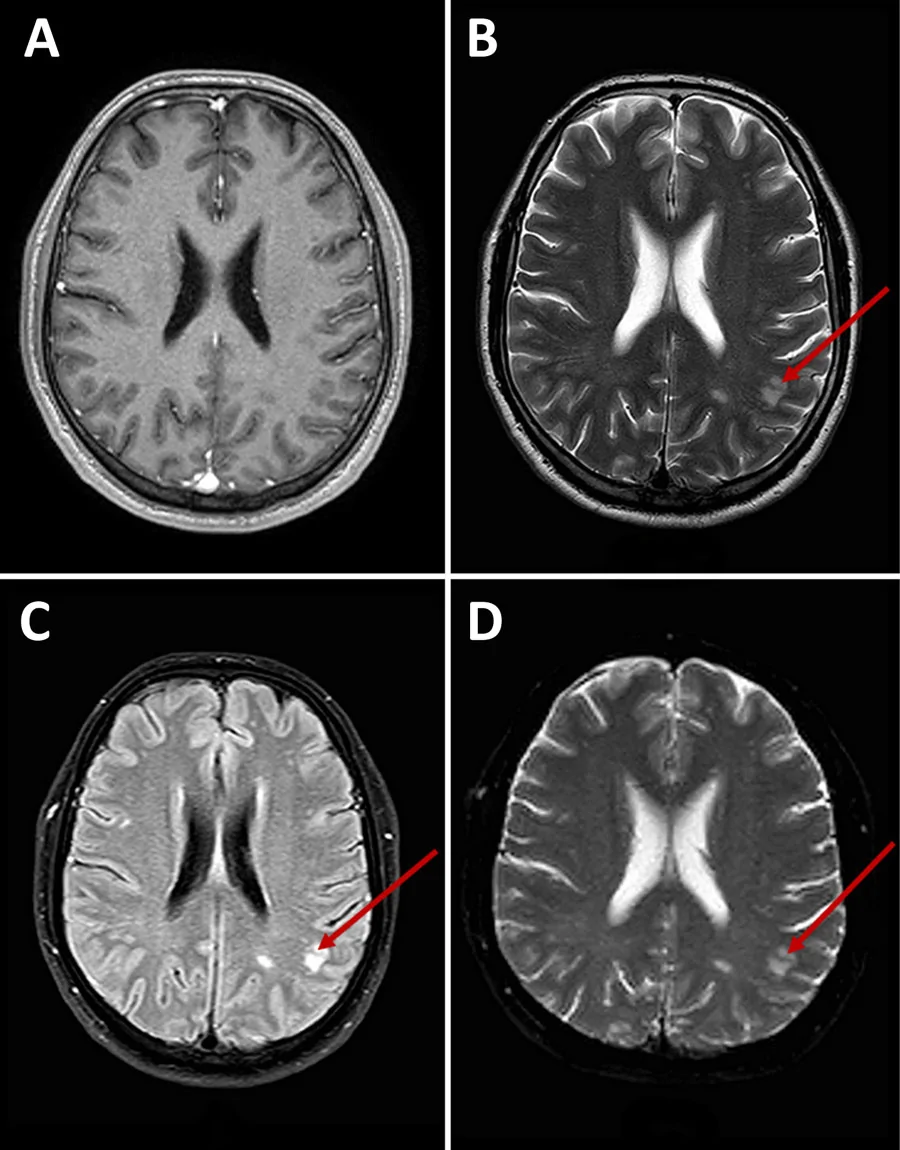
Axial brain magnetic resonance imaging of a patient with *Chlamydia psittaci* meningitis, Chile, 2023. A) T1-weighted image shows unremarkable appearance. B) T2-weighted image; C) fluid-attenuated inversion recovery image; and D) diffusion-weighted image show bilateral white matter hyperintensities (arrows).

We treated the patient empirically with intravenous ceftriaxone, ampicillin, dexamethasone, and acyclovir. We discontinued acyclovir after CSF PCR testing for HSV-1 and HSV-2 yielded negative results. We tested CSF samples by quantitative real-time PCR for parvovirus B19, *Brucella spp*., *Leptospira spp*., and *C. psittaci*. We tested serum samples serologically for *Coxiella burnetii*, *Brucella spp*., *Leptospira spp*., and *C. psittaci* (Table). We performed all analyses at the national reference laboratory of the Institute of Public Health of Chile (Santiago, Chile).

**Table:** Molecular and serologic diagnostic testing results for a study of *Chlamydia psittaci* meningitis, Chile, 2023*

Pathogen	Specimen	Method	Target or antibody	Result
Parvovirus B19	CSF	qPCR	NS1, VP1	Negative
Multiplex meningitis/encephalitis panel†	CSF	Real-time multiplex PCR assay	Pathogen-specific nucleic acid targets	Negative
*Brucella* spp.	CSF	qPCR	*bcsp31*, *IS711*	Negative
*Leptospira* spp.	CSF	qPCR	*lipL32* or 16S; specify	Negative
*Chlamydia psittaci*	CSF	qPCR	*IncA* and *CPSIT_0607*	Positive
*Toxoplasma gondii*	CSF	qPCR	*B1*, REP-529	Negative
*Coxiella burnetii*	Serum	Serology IFA	Phase I and II for IgG, IgM	Negative
*Brucella* spp.	Serum	Serology ELISA	Rose Bengal, SAT, ELISA; specify	Negative
*Leptospira* spp.	Serum	Serology ELISA	IgM	Negative
*C. psittaci*	Serum	Serology IFA	IgG, IgM	Positive IgM and IgG

*Testing performed on CSF and serum samples for routine and zoonotic pathogens. *B1*, 35-fold repetitive B1 gene of *Toxoplasma gondii*; *bcsp31*, 31 kDa *Brucella* immunogenic protein gene; *CPSIT_0607*, DNA sequence gene to *C. psittaci* hypothetical protein; CSF, cerebral spinal fluid; IFA, indirect immunofluorescence assay; *IncA*, inclusion membrane protein A; *IS711*, insertion sequence 711 of *Brucella* genus; *lipL32*, recombinant leptospiral outer membrane protein gene; NS1, nonstructural protein 1; qPCR, quantitative PCR; REP-529, noncoding 529-bp DNA fragment that is repeated 200–300 times; SAT, standard agglutination test; VP1, viral protein 1. †Allplex Meningitis-V1 and Meningitis-V2 Assays (Seegene Inc., https://www.seegene.com).

Quantitative real-time PCR of CSF returned a positive result for *C. psittaci*, as did serologic testing by indirect immunofluorescence assay, with an IgG titer of 1:128 (positive cutoff >1:64) and an IgM titer of 1:10 (positive cutoff >1:10) (Table). The patient remained stable, without neurologic deficits. Follow-up brain magnetic resonance imaging on August 26, 2023, showed no pathologic findings. The patient completed a 7-day course of intravenous antibiotics followed by 7 days of oral levofloxacin. Outpatient follow-up confirmed full recovery.

Although human psittacosis has been previously reported in Chile, including a confirmed case in 1956, a family cluster in 1984, and a 15-case outbreak in 2021 in southern regions (*6*), none of those cases involved CNS manifestations. Our case expands the geographic recognition of *C. psittaci* meningitis and highlights the need to consider zoonotic pathogens in patients with atypical CNS infections in Latin America.

Meningitis caused by *C. psittaci* is exceedingly rare. To clarify the spectrum and rarity of CNS infection caused by *C. psittaci*, we conducted a PRISMA 2020 (*7*)–guided systematic review of published cases (Appendix). We identified only 6 previously published cases of molecularly confirmed *C. psittaci* CNS infection worldwide. Because we applied stringent eligibility criteria, requiring molecular confirmation in cerebrospinal fluid or CNS tissue, we excluded most historical reports that were diagnosed solely on the basis of serology or clinical findings (Appendix Table 1), leaving only a small number of well-characterized cases for comparison. According to those reports, clinical manifestations ranged from isolated meningitis to severe encephalitis with status epilepticus or cerebral vasculitis, whereas definitive diagnosis relied almost exclusively on molecular detection in cerebrospinal fluid (Appendix Table 2). Our findings emphasize both the exceptional rarity of confirmed CNS infection and the pivotal role of molecular diagnostics in recognizing this uncommon manifestation. We identified 6 reported cases diagnosed using molecular methods, 1 from the United States (*8*), 4 from China (*9*–*12*), and 1 from Austria (*13*), and compared those with our case from Chile (Appendix).

Diagnosis of psittacosis is challenging because *C. psittaci* cannot be detected by routine bacterial cultures. Serologic testing is more widely available but often requires paired samples, which might delay confirmation and can have cross-reactivity with other *Chlamydia* species. In contrast, PCR performed on specimens collected early in the course of illness provides the highest sensitivity and specificity (*14*,*15*). Metagenomic sequencing could be a complementary diagnostic tool for characterizing *C. psittaci* CNS infections, although its availability remains limited in many clinical settings. Diagnosis in this case was difficult because of nonspecific clinical findings and the absence of classic meningeal signs. Standard CSF panels and cultures were negative, highlighting the need for molecular testing and serologic investigation in patients with a compatible exposure history and negative routine workups.

## Conclusion

Our case reinforces the need to consider zoonotic pathogens in rural and agricultural populations, particularly in Latin America, where psittacosis is underdiagnosed and potentially underreported. The favorable outcome also suggests that early empirical treatment with broad-spectrum antimicrobials, followed by targeted therapy, can be an effective approach. Doxycycline is the preferred antimicrobial agent for treating psittacosis (*3*,*4*), and 21-day duration is preferred in CNS involvement (*9*). Our case also highlights the diagnostic value of integrating epidemiologic history with molecular testing in evaluating atypical meningitis and emphasizes the importance of considering *C. psittaci* as a cause of CNS infection. 

Appendix Additional information for *Chlamydia psittaci* meningitis, Chile, 2023.
