# Supplementary material for: Chlamydia psittaci Meningitis, Chile, 2023
Source: Emerg Infect Dis. 2026 Oct;32(10):1726–9. doi: 10.3201/eid3210.251985 (PMC13632875; doi:10.3201/eid3210.251985)
Supplement: Supplementary file 1 [file 25-1985-Techapp-s1.pdf]

Article DOI: http://doi.org/10.3201/eid3210.251985

*EID cannot ensure accessibility for supplementary materials supplied by authors. Readers who have difficulty accessing supplementary content should contact the authors for assistance.*

# *Chlamydia psittaci* Meningitis, Chile, 2023

## Appendix

## PRISMA 2020 methodology

### 1. Eligibility criteria

Studies were included in the main table only if they met all of the following criteria:

- Human case report or case series with individual-level data.
- Clinical syndrome compatible with meningitis, meningoencephalitis, encephalitis, severe CNS infection, or status epilepticus attributable to CNS involvement.
- Detection of *Chlamydia psittaci* in cerebrospinal fluid or CNS material by PCR, qPCR, qRT-PCR, mNGS, 16S PCR plus sequencing, or an equivalent molecular method.
- No better alternative pathogen explaining the CNS syndrome, or, if a competing pathogen was detected, the uncertainty was explicitly acknowledged.
- Sufficient case-level data to allow extraction of at least age, sex, neurologic presentation, diagnostic method, sample type, treatment, and outcome.

The following were excluded from the main comparative table:

- Reports based only on serology, clinical neurologic manifestations, or non-CNS specimens without molecular detection in CSF or CNS tissue.
- Reports of psittacosis with neurologic symptoms (cranial nerve palsies, Guillain-Barré syndrome, cerebral infarction, myeloradiculitis, reversible splenial lesion syndrome) without evidence of direct CNS infection by *C. psittaci*.
- Reports labeled as 'CNS infection' when the organism detected in CSF was not *C. psittaci*.
- Reviews, narrative summaries, or articles without original case-level data.
- Veterinary or non-human reports.

#### 2. Information sources

Two bibliographic databases were searched: PubMed/MEDLINE and Scopus, both on 30 June 2026. Supplementary sources included backward citation (reference-list) screening of all records meeting eligibility criteria, and consultation with co-authors, which identified additional records not captured by either database search (Newton et al. 1996, Punter et al. 2010, Gao et al. 2025, Tian et al. 2025, Denmark JC 1957, Hughes et al. 1995). LILACS/BVS (Virtual Health Library, Latin America and Caribbean) was searched on the same date using the same conceptual terms adapted to the BVS interface, returning 10 records. Eight were duplicates already captured by PubMed or Scopus, plus two within-LILACS duplicates of the same article. The two unique new records identified were: del Castillo M & Jáuregui Rueda H (Medicina B Aires, 1987), describing non-pulmonary manifestations of psittacosis in Argentina (excluded: serology-based, pre-molecular era); and Guilbride PDL (West Indian Med J, 1953), a Caribbean public health review (excluded at title/abstract: wrong study type). Del Castillo 1987 article is notable as the only Latin American report of extrapulmonary psittacosis identified in any of the three databases searched; its exclusion under strict criteria further supports the novelty of the present case.

#### 3. Search strategy

The following search string was used in PubMed/MEDLINE on 30 June 2026 (no date or language filters):

*(“Chlamydia psittaci”[Title/Abstract] OR “Chlamydophila psittaci”[Title/Abstract] OR “psittacosis”[Title/Abstract]) AND (“meningitis”[Title/Abstract] OR “encephalitis”[Title/Abstract] OR “meningoencephalitis”[Title/Abstract] OR “central nervous system”[Title/Abstract] OR “cerebrospinal fluid”[Title/Abstract] OR “CSF”[Title/Abstract] OR “status epilepticus”[Title/Abstract] OR “neurological”[Title/Abstract])*

This search returned 33 records.

The following equivalent string was used in Scopus on 30 June 2026 (Title/Abstract field, no date or language filters):

*TITLE-ABS(“Chlamydia psittaci” OR “Chlamydophila psittaci”) AND TITLE-ABS(“cerebrospinal fluid” OR “CSF” OR “meningitis” OR “encephalitis” OR “meningoencephalitis” OR “status epilepticus”)*

This Scopus search returned 40 records. Reference lists of all records meeting eligibility criteria were manually screened for additional relevant reports. Co-author consultation identified 6 further

records not captured by either database (Newton et al. 1996, Punter et al. 2010, Gao et al. 2025, Tian et al. 2025, Denmark JC 1957, Hughes et al. 1995). After removing 23 duplicates between PubMed and Scopus, 56 unique records were screened.

**4. Study selection process**

All 56 unique records (33 from PubMed + 40 from Scopus − 23 duplicates + 6 additional sources) were screened by title/abstract against the eligibility criteria. Thirty-two records were excluded at this stage for clearly wrong population (animal-only studies), wrong organism (*C. trachomatis, C. pneumoniae*), wrong study type (basic science, genomic characterizations, general reviews, wildlife surveillance studies), or irrelevant topic. The remaining 24 records were assessed at full text. Eighteen records were excluded under the strict CNS-molecular criteria (Appendix Table 1). Six studies met all eligibility criteria and were included. The present Chilean case was not counted as a case identified through the bibliographic search; it was included separately in the comparative table.

**5. Data extraction items**

For each included study, the following variables were extracted and tabulated in Appendix Table 2: country and year of publication; patient age and sex; epidemiologic exposure; main clinical syndrome; initial presentation; neurologic manifestations; meningeal signs; brain imaging findings; CSF cell count, protein, and glucose; routine CSF microbiology results; diagnostic method for C. psittaci and specimen type; orthogonal confirmation; competing pathogens; respiratory/systemic involvement; antibiotic treatment; ICU admission; mechanical ventilation; clinical outcome; proposed certainty category for CNS involvement; and key limitations. Data were extracted from published full texts or, where full text was unavailable, from the published abstract with explicit notation.

**6. Results of the search**

PubMed/MEDLINE (30 June 2026) returned 33 records; Scopus (30 June 2026) returned 40 records. After removal of 23 duplicates between the two databases, 50 unique records were obtained from database searches. Six additional records were identified through reference-list screening and co-author consultation, for a total of 56 unique records screened. Thirty-two records were excluded at title/abstract screening (animal studies, wrong organism, wrong study type). Of the 24 full-text articles assessed, 18 were excluded under the strict CNS-molecular criteria (Table 1). Six studies met all inclusion criteria: Walder et al. (2003, Austria), Shi et al. (2021, People's Republic of China), Yin et al. (2021, People's Republic of China), Davar et al. (2021, United States), Zhang et al. (2024, People's Republic of China), and Zhao et al. (2026, People's Republic of China). Combined with the present Chilean case, 7 cases are compared in the main comparative table (Appendix Tables 2 and 3).

The Scopus search identified five records not captured by PubMed (Cazenave-Roblot et al. 1988, Henrion et al. 2002, Korman et al. 1997, Kanerva et al. 2013, Ragnaud et al. 1985). All five were assessed at full text and excluded under the strict CNS-molecular criteria (Table). The LILACS/BVS search did not identify additional records of *C. psittaci* meningitis from Latin America.

Three cases (Walder 2003, Davar 2021, present case) were classified as 'confirmed–strong' based on molecular detection in CSF with orthogonal confirmation (sequencing, confirmatory PCR, or positive serology). Three additional cases (Shi 2021, Zhang 2024, Zhao 2026) were classified as 'probable–high' based on mNGS detection in CSF without additional independent confirmation. One case (Yin 2021) was classified as 'probable with coinfection caveat' because competing *Streptococcus pneumoniae* reads were also detected by mNGS.

The complete study selection process is shown in the Appendix Figure.

**References (1–6, included studies; 7–26, excluded studies [full-text assessment])**


1. Walder G, Schönherr H, Hotzel H, Speth C, Oehme A, Dierich MP, et al. Presence of *Chlamydophila psittaci* DNA in the central nervous system of a patient with status epilepticus. Scand J Infect Dis. 2003;35:71–3. PubMed https://doi.org/10.1080/00365540210000026984

2. Shi Y, Chen J, Shi X, Hu J, Li H, Li X, et al. A case of *Chlamydia psittaci* caused severe pneumonia and meningitis diagnosed by metagenome next-generation sequencing and clinical analysis: a case report and literature review. BMC Infect Dis. 2021;21:621. PubMed https://doi.org/10.1186/s12879-021-06205-5

3. Yin XW, Mao ZD, Zhang Q, Ou QX, Liu J, Shao Y, et al. Clinical metagenomic sequencing for rapid diagnosis of pneumonia and meningitis caused by *Chlamydia psittaci.* World J Clin Cases. 2021;9:7693–703. PubMed https://doi.org/10.12998/wjcc.v9.i26.7693

4. Davar K, Wilson MR, Miller S, Chiu CY, Vijayan T. A rare bird: diagnosis of psittacosis meningitis by clinical metagenomic next-generation sequencing. Open Forum Infect Dis. 2021;8:ofab555. PubMed https://doi.org/10.1093/ofid/ofab555

5. Zhang W, Zhang S, Huang X, Dai L, Gao Y, Cao H, et al. A case of *Chlamydophila psittaci* caused pneumonia and encephalitis diagnosed by metagenome next-generation sequencing and clinical analysis. Jundishapur J Microbiol. 2024;17:e144399. https://doi.org/10.5812/jjm-144399

6. Zhao C, Liu Y, Li J. Radiological features of encephalitis of Chlamydia psittaci. Neurol India. 2026;74:378–9. PubMed https://doi.org/10.4103/neurol-india.Neurol-India-D-25-00440

7. Denmark JC. Encephalitis due to the psittacosis-lymphogranuloma venereum group of viruses; a case of ornithosis encephalitis. BMJ. 1957;2:1531. PubMed https://doi.org/10.1136/bmj.2.5060.1531

8. Cazenave-Roblot F, Fort E, Breux JP, Neau JP, Castets M, Becq-Giraudon B. Ornithosis and central nervous system involvement: a case report [in French]. Med Mal Infect. 1988;18:391–3. https://doi.org/10.1016/S0399-077X(88)80185-9

9. Hughes P, Chidley K, Cowie J. Neurological complications in psittacosis: a case report and literature review. Respir Med. 1995;89:637–8. PubMed https://doi.org/10.1016/0954-6111(95)90236-8

10. Saeki S, Hirata I, Fukusako T, Negoro K, Nogaki H, Morimatsu M. A case of psittacosis with psychiatric symptoms, abnormal EEG, and abnormal SPECT [in Japanese]. No To Shinkei. 1996;48:1141–1145. PubMed

11. Reis J, Le Faou A, Levy F, Kapfer MT, Gut JP. Confusional form of Chlamydia psittaci encephalitis. Diagnostic value of microimmunofluorescence. A case [in French]. Presse Med. 1985;14:87–89. PubMed

12. Ragnaud JM, Dupon M, Echinard E, Lacut JY, Aubertin J. Aspects actuels des infections à Chlamydia psittaci. À propos de 32 observations. Med Mal Infect. 1985;15:706–13. https://doi.org/10.1016/S0399-077X(85)80082-2

13. Newton P, Lalvani A, Conlon CP. Psittacosis associated with bilateral 4th cranial nerve palsies. J Infect. 1996;32(1):63-65. PubMed https://doi.org/10.1016/s0163-4453(96)80011-9

14. Korman TM, Turnidge JD, Grayson ML. Neurological complications of chlamydial infections: case report and review. Clin Infect Dis. 1997;25:847–51. PubMed https://doi.org/10.1086/515536

15. Ionescu AM, Khare D, Kavi J. Birds of a feather: an uncommon cause of pneumonia and meningoencephalitis. BMJ Case Rep. 2016;2016:bcr2016216879. PubMed https://doi.org/10.1136/bcr-2016-216879

16. Henrion E, Trippaerts M, Lepage P. Severe multiorgan psittacosis in a 10-year-old boy [in French]. Arch Pediatr. 2002;9:810–3. PubMed https://doi.org/10.1016/S0929-693X(01)00993-9

17. Punter MNM, Varma AR. Myeloradiculitis with meningoencephalopathy and optic neuritis in a case of previous *Chlamydia psittaci* infection. BMJ Case Rep. 2010;2010:bcr1220092521. PubMed https://doi.org/10.1136/bcr.12.2009.2521

18. Kanerva M, Nissinen J, Moilanen K, Mäki M, Lahdenne P, Pitkäranta A. Microbiologic findings in acute facial palsy in children. Otol Neurotol. 2013;34:e82–7. PubMed https://doi.org/10.1097/MAO.0b013e318289844c

19. Wang J, Dong S, Fang M, Fan Z, Xu Y. Omadacycline for the treatment of severe *Chlamydia psittaci* pneumonia complicated with Guillain-Barré syndrome. Infect Drug Resist. 2024;17:81–7. PubMed https://doi.org/10.2147/IDR.S437100

20. Branley JM, Weston KM, England J, Dwyer DE, Sorrell TC. Clinical features of endemic community-acquired psittacosis. New Microbes New Infect. 2014;2:7–12. PubMed https://doi.org/10.1002/2052-2975.29

21. Ni Y, Zhong H, Gu Y, Liu L, Zhang Q, Wang L, et al. Clinical features, treatment, and outcome of psittacosis pneumonia: a multicenter study. Open Forum Infect Dis. 2023;10:ofac518. PubMed https://doi.org/10.1093/ofid/ofac518

22. Cizman M, Jazbec J. Etiology of acute encephalitis in childhood in Slovenia. Pediatr Infect Dis J. 1993;12:903–8. PubMed https://doi.org/10.1097/00006454-199311000-00002

23. Gao J, Lao C, Chen J. Chlamydia psittacosis infection complicating cerebral infarction: a case report and literature review. Medicine (Baltimore). 2025;104:e44854. PubMed https://doi.org/10.1097/MD.0000000000044854

24. Tian S, Xiao Y, Dong C. Psittacosis combined with central nervous system infection and acute cerebral infarction: a case report based on metagenomic next-generation sequencing. Neurologist. 2025;30:303–6. PubMed https://doi.org/10.1097/NRL.0000000000000636

25. del Castillo M, Jáuregui Rueda H. Non-pulmonary manifestations of human psittacosis [in Spanish]. Medicina (B Aires). 1987;47:322–4. PubMed

26. Guilbride PDL. The importance of animal disease to public health in the Caribbean with special reference to psittacosis. West Indian Med J. 1953;2:11–36.

**Appendix Table 1.** Full-text articles excluded after full-text assessment and reasons for exclusion from a study of *Chlamydia psittaci* meningitis, Chile, 2023.

| Reference | Original reason for consideration | Decision | Reason for exclusion (strict CNS criteria) |
|---|---|---|---|
| Denmark JC, 1957 | Historical ornithosis encephalitis | Excluded | Historical report; no modern molecular confirmation in CSF or CNS tissue. |
| Cazenave-Roblot F et al Med Mal Infect; 1988;18:391-3 | Ornithosis with CNS involvement (cerebellar syndrome, urinary retention) | Excluded | CSF reported as normal; diagnosis based on serology only; no molecular detection in CSF. |
| Reis J et al., Presse Med;14 (2):87:1985 | Confusional encephalitis attributed to *C. psittaci* | Excluded | Diagnosis based on microimmunofluorescence serology only; no molecular detection in CSF or CNS material. |
| Saeki S et al., No To Shinkei;48:1141:1996 | Psittacosis with psychiatric symptoms, abnormal EEG/SPECT | Excluded | Neurologic syndrome described; no CNS molecular evidence of *C. psittaci*; full text in Japanese. |
| Henrion E et al., Arch Pediatr;9 (8):810 2002 | Severe psittacosis with multiorgan failure in a 10year-old boy; meningism on admission | Excluded | Meningism reported clinically but diagnosis based on serology; no molecular detection in CSF or CNS material. |
| Newton P et al., J Neurol Neurosurg Psychiatry 1996 | Cranial nerve palsies associated with psittacosis | Excluded | Neurologic complication of psittacosis; *C. psittaci* not detected in CSF or CNS tissue. |
| Korman TM et al., Clin Infect Dis;25 (4):847 1997 | Neurologic complications of Chlamydial infections: case report and review | Excluded | Primary case involves *C. pneumoniae*, not *C. psittaci;* historical *C. psittaci* neurologic cases cited in review are serology-based only. |
| Punter MT et al., (case report) 2010 | Myeloradiculitis/meningoencephalopathy after psittacosis | Excluded | Post-infectious or para-infectious neurologic syndrome; no direct detection of *C. psittaci* in CSF or CNS tissue. |
| Kanerva M et al., Otol Neurotol;34 (7) 2013 | Microbiologic findings in acute facial palsy in children | Excluded | Cranial nerve palsy; neurologic complication of *C. psittaci*, not demonstrable CNS infection; *C. psittaci* not detected in CSF. |
| Wang J et al., Infect Drug Resist;17 2024 | *C. psittaci* pneumonia complicated with Guillain-Barré syndrome | Excluded | Guillain-Barré syndrome = peripheral immune-mediated complication; *C. psittaci* not detected in CSF or CNS tissue. |
| Gao J et al., 2025 | Psittacosis complicated by cerebral infarction | Excluded | Cerebrovascular complication of systemic psittacosis; *C. psittaci* not detected in CNS. |
| Ionescu AM et al., 2016 BMJ Case Rep; PMID 27620382 | Pneumonia and meningoencephalitis | Excluded main table | *C. psittaci* DNA detected in sputum, not in CSF or CNS material. May be cited narratively as probable neurologic involvement but does not meet strict criterion 3. |
| Tian S et al., Neurologist. 2025 Sep 1;30 (5):303–306. | Psittacosis with 'CNS infection' and cerebral infarction | Excluded | Available description suggests the pathogen detected in CSF was not *C. psittaci*; therefore, not evidence of *C. psittaci* CNS invasion. |
| Branley JM et al., 2014 New Microbes New Infect;2:7 | Clinical features of endemic community-acquired psittacosis | Excluded | Respiratory psittacosis cohort; no CNS detection of *C. psittaci* reported. |
| Ni Y et al., 2023 Open Forum Infect Dis;10:ofac518 | Multicenter psittacosis pneumonia cohort | Excluded | Respiratory psittacosis cohort; no CNS detection of *C. psittaci* reported. |
| Cizman M & Jazbec J, 1993 Pediatr Infect Dis J;12:903 | Pediatric cohort; *C. psittaci* among etiologies | Excluded | Cohort-level serology; no individual-level CNS molecular data; criterion 4 (individual data) not met. |
| Ragnaud JM et al., Med Mal Infect;15(11):1985 | Current aspects of *C. psittaci* infections: 32 cases | Excluded | Large case series from the pre-molecular era; diagnosis based on serology; no individual-level CNS molecular data. |
| del Castillo M & Jáuregui Rueda H, 1987 Medicina (B.Aires);47(3):322 | Non-pulmonary manifestations of human psittacosis; case series, Argentina | Excluded | Diagnosis based on serology; pre-molecular era (1987); no molecular detection in CSF or CNS. Identified only in LILACS/BVS — the only Latin American case report on extrapulmonary psittacosis found in any of the three databases searched. |
| Hughes P et al., 1995 Respir Med;89:637 | Narrative review of neurologic complications | Excluded | Narrative review without primary case-level data; criterion 5 not met. |

**Appendix Table 2.** Characteristics of published cases with molecularly confirmed *Chlamydia psittaci* central nervous system infection identified through the PRISMA-guided systematic review, compared with the present case, for a study of *Chlamydia psittaci* meningitis, Chile, 2023.

| Characteristic | Present case, Chile, 2023 | Walder et al., Austria, 2003 | Shi et al., People's Republic of China, 2021 | Yin et al., People's Republic of China, 2021 | Davar et al., United States, 2021 | Zhang et al., People's Republic of China, 2024 | Zhao et al., People's Republic of China, 2026 |
|---|---|---|---|---|---|---|---|
| Age / sex | 50-y-old man | 62-y-old man | 54-y-old woman | 5-y-old boy | 67-y-old man | 72 y old woman | 37-y-old man |
| Epidemiologic exposure | Farm worker; exposure to birds and livestock | Parrot owner | Denied direct bird, poultry, or horse contact | Exposure to birds | Travel to Sicily; parrot sightings | Not described | Not reported |
| Main clinical syndrome | Meningitis | Status epilepticus / neuroornithosis | Severe pneumonia with meningitis | Pneumonia and meningitis | Meningitis | Severe pneumonia with encephalitis (cerebral vasculitis) | Encephalitis (progressive frontotemporal/insular masslike lesion) |
| Initial presentation | Fever, myalgia, chills, headache, odynophagia | Fatigue, myalgia, sudden high fever | Headache, fever, dyspnea | Fever, dizziness, vomiting | Fever, night sweats, arthralgias, cough | Respiratory and neurologic symptoms | One month of seizures and unconsciousness |
| Neurologic manifestations | Persistent headache; no focal deficits | Generalized status epilepticus | Severe headache; memory loss | Seizures; meningitis manifestations | Neck stiffness; altered mental status | Headache, altered consciousness | Seizures, coma, progressive bilateral encephalitic lesions |
| Meningeal signs | Absent | Not specified | Not specified | Not specified | Present | Not reported | Not reported |
| Brain imaging | MRI: transient white matter hyperintensities (T2/FLAIR/DWI); follow-up normal | Not fully reported | Not reported | Not reported | Not reported | Cerebrovascular/vasculitis changes (limited data) | MRI: focal left frontal lesion progressing to bilateral frontotemporal and insular involvement |
| CSF cells (cells/mm$^3$) | 1,033 | Normal (no pleocytosis) | 6 | Not reported | 373 | Mild, 7 cells/µL | Not reported |
| CSF protein | 1.21 g/L | 0.93 g/L | 0.267 g/L (normal) | Not reported | 2.22 g/L | 0.391 g/L | 0.182 g/L |
| CSF glucose | 50.1 mg/dL | Not reported | Not reported | Not reported | Not reported | 3.48 mmol/L | 4.37 mmol/L |
| Routine CSF microbiology | Gram stain and multiplex meningitis/encephalitis PCR panel negative | Not reported | Not reported | Not reported | Not reported | Not reported | Not reported |
| Diagnostic method for *C. psittaci* | qPCR in CSF (IncA, CPSIT_0607) plus serology (IFA) | PCR and 16S sequencing in CSF | mNGS in CSF and BALF | mNGS in CSF | mNGS in CSF plus confirmatory CDC PCR in CSF | mNGS in blood and CSF | mNGS in CSF |
| Positive specimen | CSF (qPCR); serum (serology) | CSF | CSF and BALF | CSF | CSF | Blood and CSF | CSF |
| Orthogonal confirmation | Serology positive (IgG 1:128; IgM 1:10) | Molecular sequencing | Not clearly reported | Not clearly reported; competing organism detected | Confirmatory CDC PCR in CSF | Not reported | Not reported (no confirmatory PCR or serology) |
| Competing pathogens | Routine tests negative; multiplex panel negative | Not reported | No other relevant pathogen reported | *Streptococcus pneumoniae* reads also detected by mNGS | Not reported | Not reported | No other likely pathogens identified |

| Characteristic | Present case, Chile, 2023 | Walder et al., Austria, 2003 | Shi et al., People's Republic of China, 2021 | Yin et al., People's Republic of China, 2021 | Davar et al., United States, 2021 | Zhang et al., People's Republic of China, 2024 | Zhao et al., People's Republic of China, 2026 |
|---|---|---|---|---|---|---|---|
| Respiratory / systemic involvement | Mild systemic syndrome; no severe pneumonia | Systemic illness; no severe pneumonia described | Severe pneumonia; ARDS; mechanical ventilation | Pneumonia | Respiratory and systemic symptoms; no ARDS reported | Severe pneumonia | No pneumonia described in brief report |
| Treatment | Ceftriaxone/ampicillin (IV) → levofloxacin (oral, 7 d) | Doxycycline for 30 d | Doxycycline, moxifloxacin, azithromycin | Azithromycin, rifampin, vancomycin | Levofloxacin plus ampicillin/TMP-SMX | Azithromycin and Piperacillin/tazobactam switched to doxycycline for 10 d | Empirical: acyclovir, cefoperazone, methylprednisolone, IVIG; targeted doxycycline not clearly before death |
| ICU admission | No | Yes | Yes (prolonged course) | Not reported | Not reported | Yes | Yes |
| Mechanical ventilation | No | Yes (44 d) | Yes | Not reported | Not reported | Not reported | Not reported |
| Clinical outcome | Complete recovery | Slow recovery | Complete recovery | Complete recovery after targeted therapy | Complete recovery | Improved, discharged | Death |
| Proposed certainty of CNS involvement | Confirmed–strong (qPCR in CSF + serology + negative routine workup) | Confirmed–strong (PCR/16S sequencing in CSF) | Probable–high (mNGS in CSF; low CSF cell count) | Probable with coinfection caveat (mNGS in CSF; competing *S. pneumoniae* reads) | Confirmed–strong (mNGS + confirmatory CDC PCR in CSF) | Probable–high (mNGS in blood and CSF; no confirmatory PCR or serology reported) | Probable–high (mNGS in CSF; no confirmatory PCR, no serology, no read counts reported) |
| Key limitation | Single case; rarity of pathogen; reproducibility depends on molecular assay details | Historical molecular methods; limited extractable data | Low CSF cell count and mild CSF inflammation; low molecular signal | Competing S. pneumoniae detection; coinfection vs. coincidental detection unclear | No major limitation for CNS attribution | Limited published data; incomplete clinical description, no PCR or serology performed | Brief report; no read counts, no confirmatory PCR, no serology; antimicrobial course before death unclear |

Abbreviations: ARDS, acute respiratory distress syndrome; BALF, bronchoalveolar lavage fluid; CDC, Centers for Disease Control and Prevention; CNS, central nervous system; CSF, cerebrospinal fluid; IFA, immunofluorescence assay; IVIG, intravenous immunoglobulin; mNGS, metagenomic next-generation sequencing; PCR, polymerase chain reaction; qPCR, quantitative PCR; TMP-SMX, trimethoprim-sulfamethoxazole.

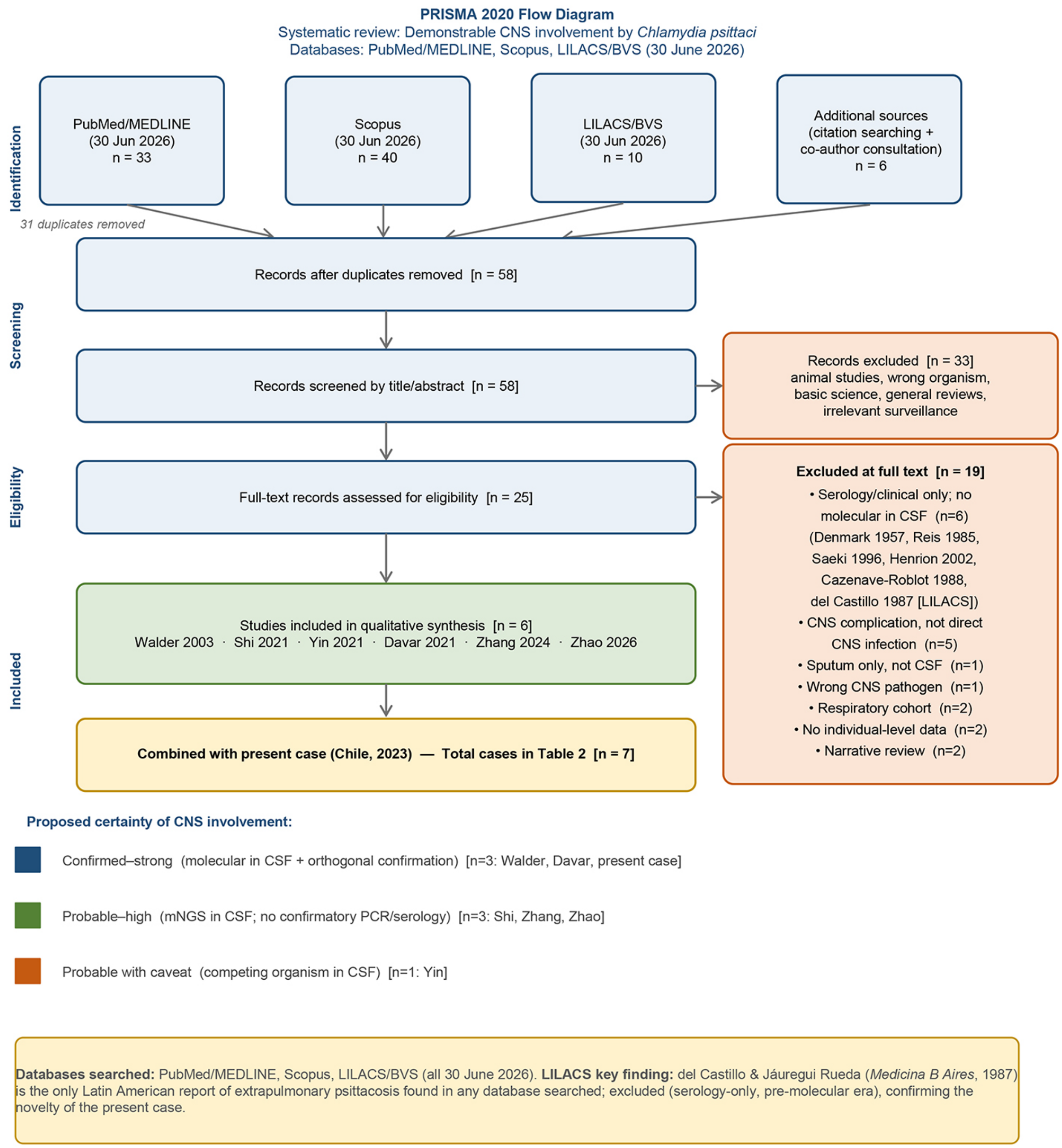


**Appendix Figure.** PRISMA 2020 flow diagram for a study of *Chlamydia psittaci* meningitis, Chile, 2023.
